# Erratum

**DOI:** 10.5152/TJAR.2022.090222

**Published:** 2022-02-01

ErratumIn the article by Şensoy et al. entitled “Effect of Isoflurane Exposure with Administration of Polyunsaturated Fatty Acids on Cognition in Developing Rats” published in the December 2020 issue of Turkish Journal of Anaesthesiology & Reanimation (Turk J Anaesthesiol Reanim 2020; 48: 477-483, DOI: 10.5152/TJAR.2020.128), authors declared that the ORCID ID of Didem Aldemir Şensoy’s erroneously written wrong. 

Author’s correction request were evaluated and accepted by the Editorial Board. Thus, the article has been ­corrected accordingly and updated in the journal’s archive. You may access the updated article via the link below.

https://turkjanaesthesiolreanim.org/en/effect-of-isoflurane-exposure-with-administration-of-polyunsaturated-fatty-acids-on-cognition-in-developing-rats-13807

